# ATP-Binding Cassette (ABC) Transporters of the Human Respiratory Tract Pathogen, *Moraxella catarrhalis*: Role in Virulence

**DOI:** 10.1371/journal.pone.0158689

**Published:** 2016-07-08

**Authors:** Timothy F Murphy, Aimee L. Brauer, Antoinette Johnson, Charmaine Kirkham

**Affiliations:** 1 Clinical and Translational Research Center, University at Buffalo, the State University of New York, Buffalo, NY, United States of America; 2 Division of Infectious Diseases, Department of Medicine, University at Buffalo, the State University of New York, Buffalo, NY, United States of America; 3 Department of Microbiology, University at Buffalo, the State University of New York, Buffalo, NY, United States of America; University of Toledo College of Medicine and Life Sciences, UNITED STATES

## Abstract

*Moraxella catarrhalis* is a human respiratory tract pathogen that causes otitis media (middle ear infections) in children and respiratory tract infections in adults with chronic obstructive pulmonary disease. In view of the huge global burden of disease caused by *M*. *catarrhalis*, the development of vaccines to prevent these infections and better approaches to treatment have become priorities. In previous work, we used a genome mining approach that identified three substrate binding proteins (SBPs) of ATP-binding cassette (ABC) transporters as promising candidate vaccine antigens. In the present study, we performed a comprehensive assessment of 19 SBPs of 15 ABC transporter systems in the *M*. *catarrhalis* genome by engineering knockout mutants and studying their role in assays that assess mechanisms of infection. The capacity of *M*. *catarrhalis* to survive and grow in the nutrient-limited and hostile environment of the human respiratory tract, including intracellular growth, account in part for its virulence. The results show that ABC transporters that mediate uptake of peptides, amino acids, cations and anions play important roles in pathogenesis by enabling *M*. *catarrhalis* to 1) grow in nutrient-limited conditions, 2) invade and survive in human respiratory epithelial cells and 3) persist in the lungs in a murine pulmonary clearance model. The knockout mutants of SBPs and ABC transporters showed different patterns of activity in the assay systems, supporting the conclusion that different SBPs and ABC transporters function at different stages in the pathogenesis of infection. These results indicate that ABC transporters are nutritional virulence factors, functioning to enable the survival of *M catarrhalis* in the diverse microenvironments of the respiratory tract. Based on the role of ABC transporters as virulence factors of *M*. *catarrhalis*, these molecules represent potential drug targets to eradicate the organism from the human respiratory tract.

## Introduction

*Moraxella catarrhalis* is an exclusively human pathogen that is a common cause of otitis media (middle ear infections) in children and respiratory tract infections in adults with chronic obstructive pulmonary disease [1–4]. The enormous global morbidity caused by *M*. *catarrhalis* is driving research in understanding mechanisms of pathogenesis of infection in order to guide drug and vaccine development to better treat and prevent infections by this pathogen. To identify vaccine antigens for *M*. *catarrhalis*, we used a genome mining approach to predict genes that encode proteins that are expressed on the bacterial surface and then assessed selected proteins as putative vaccine antigens [5]. This approach led to the discovery of three substrate binding proteins (SBPs) of ATP-binding cassette (ABC) transporters that are promising vaccine antigens that are now in various stages of development [6–8]. This work has led to more comprehensive studies of substrate-binding proteins of ABC transporter systems in the *M*. *catarrhalis* genome that we report here.

ABC transporters are ubiquitous in nature. In eukaryotes, they consist of two transmembrane permease domains and two ATPase domains and function primarily in mediating efflux of molecules. By contrast, in bacteria and archaea, ABC transporters generally have one or more SBPs in addition to permeases and ATPases, and function predominantly in uptake of molecules as opposed to efflux [9, 10]. ABC transporters transport molecules against a chemical gradient driven by free energy change associated with ATP hydrolysis. The SBPs bind a wide range of molecules, including ions, amino acids, peptides, lipoproteins and carbohydrates, and are key determinants of substrate specificity of ABC transport systems.

This functional diversity of SBPs is reflected in large sequence diversity among SBPs as well. In spite of little primary amino acid sequence homology among SBPs with differing binding specificities, SBPs have a common mixed α-helix/β-sheet structure with two domains linked by a hinge with the substrate binding occurring in the region between these two domains [11, 12].

ABC transporters comprise up to 5% of bacterial genomes and have critical functions in uptake of nutrients and other molecules [13, 14]. In addition, bacterial SBPs and ABC transporters play key roles in the pathogenesis of infection facilitating pathogenic mechanisms that include biofilm formation, adherence to and invasion of host cells, intracellular survival and nasopharyngeal colonization [15–18]. For example, OppA, the SBP of the oligopeptide permease ABC transporter is a nutritional virulence factor for respiratory tract infection caused by *M*. *catarrhalis* [19]. The role of bacterial SBPs in nutrient acquisition and in other key cellular functions, make ABC transporter systems excellent drug targets for the development of novel antimicrobial agents [20, 21]. In addition, selected SBPs have been identified and characterized as promising vaccine antigens for protection against infections caused by *Yersinia pestis*, *Neisseria meningitidis* and *M*. *catarrhalis* [6–8, 22, 23].

The goal of the present study is to perform a comprehensive study of the SBPs of ABC transporters of *M*. *catarrhalis*. We studied a total of 19 predicted SBPs in 15 predicted ABC transporter systems based on annotation of the *M*. *catarrhalis* genome [24–26]. We engineered knockout mutants and assessed the role of ABC transporters and their SBPs in growth, adherence to and invasion of human respiratory epithelial cells, and persistence in the lungs in a murine pulmonary clearance challenge model. The results identified several SBPs that play important roles in survival and virulence and, thus, represent potential targets of novel antimicrobial agents.

## Materials and Methods

### Bacterial strains and growth

*M*. *catarrhalis* strain 035E, provided by Eric Hansen, is a prototype otitis media strain that was isolated from the middle ear fluid of a child with otitis media in Dallas, TX. *M*. *catarrhalis* was grown on brain heart infusion (BHI) plates at 35°C with 5% CO_2_ or in BHI broth with shaking at 37°C. Chocolate agar was used to grow *M*. *catarrhalis* strains that were recovered from murine lungs in the mouse pulmonary clearance model. In selected growth experiments, wild type and mutant strains were grown in chemically defined media (CDM) as previously described [27, 28].

### Construction of mutants

Mutant construction was accomplished by using overlap extension PCR and homologous recombination as described previously [6, 29]. Briefly, the transforming DNA for the mutants was composed of 3 overlapping fragments that included ~1 kb upstream of the gene or gene cluster being knocked out (fragment 1), the nonpolar kanamycin resistance cassette amplified from plasmid pUC18K (fragment 2), and ~1 kb downstream of the gene (fragment 3) [30]. Mutants were constructed by transformation of strain O35E with a fragment composed of fragments 1, 2, and 3 and selection on BHI plates containing 50 μg/ml of kanamycin. The insert and surrounding sequences of each of the mutants were confirmed by sequence analysis.

### Assessment of bacterial growth

Growth curves were performed using a Bioscreen C automated growth curve analysis system (Oy Growth Curves AB, Helsinki, Finland). *M*. *catarrhalis* strains were grown in BHI broth overnight with shaking at 37°C and 225 rpm. Growth curves were performed with a 200 μl inoculum of the overnight cultures diluted in BHI broth or CDM using a dilution between 1:100 and 1:1000 depending on the growth characteristics of each mutant. In each experiment comparing wild type and mutant, the wild type and corresponding mutant were subjected to identical dilutions and growth conditions. Each growth condition was performed as five replicate wells in each experiment, with optical density measurements taken at 600 nm at 30 minute intervals at 37°C with constant shaking (machine settings: fast speed, high amplitude).

### Assessment of Adherence and Invasion of Respiratory Epithelial Cells

Quantitative adherence and invasion assays were performed with A549 cells (human type II alveolar lung epithelium; ATCC CCL85) grown in F-12K media (Gibco) plus 10% fetal bovine serum as previously described [28]. Based on initial pilot studies testing multiplicity of infection (MOI) of 1, 10 and 100, an MOI of 1 was determined to be optimal. Thus, all adherence and invasion experiments in the present study use an MOI of 1. Briefly, 2 × 10^5^ A549 cells were seeded into each well of a 24 well tissue culture plate and incubated for ~48 hours when cells showed confluent growth. Cells were inoculated with BHI broth-grown log phase bacteria and the plates were centrifuged at 170 × g for 5 minutes at room temperature to facilitate contact between bacteria and A549 cells. Plates were incubated for 3 hours at 37°C. Nonadherent cells were removed by gently washing the wells 3 times with PBS. To quantify adherent cells, 200 μl of trypsin (0.25%) was added to each well and plates were incubated at 37°C for 10 minutes to remove adherent cells. A 300 μl volume of 1% saponin was added to each well, contents were pipetted into microfuge tubes and, after vigorous vortexing, were plated in duplicate to determine bacterial cell counts. Adherence was measured as colony forming units (cfu) per ml.

In each experiment, mutants were assayed simultaneously with wild type. Results of assays with the mutants (cfu/ml) were expressed as a per cent of the result with wild type (cfu/ml) that was performed simultaneously. Each experiment was repeated 3 times and the average and standard deviation were calculated. Statistical significance was determined by performing a two-tailed t test. A p-value of ≤0.05 was considered significant.

To measure invasion, gentamicin (100 μg/ml) was added to wells after 3 hours of incubation of A549 cells with bacteria. Non adherent cells were removed by washing and wells were incubated with gentamicin for 1 hour at 37°C. Epithelial cells were recovered with trypsin and lysed with saponin as described above, and then plated in duplicate. Invasion was measured as cfu/ml. Results of assays with the mutants (cfu/ml) were expressed as a percentage of the result with wild type (cfu/ml) that was performed simultaneously. Each experiment was repeated 3 times and the average and standard deviation were calculated. Statistical significance was determined by performing a two-tailed t test. A p-value of ≤0.05 was considered significant.

### Pulmonary clearance model

The mouse pulmonary clearance model was performed as described previously [19, 28]. The study described in this manuscript was approved by the University at Buffalo Institutional Animal Care and Use Committee (Project number MED0706Y). BALB/c mice between 6 and 10 weeks of age were challenged simultaneously with the *M*. *catarrhalis* wild type strain O35E and the mutant, and clearance of the strains was assessed. Briefly, overnight cultures of wild type and the mutant were used to inoculate 50 ml of BHI broth cultures, which were then grown to log phase (Optical density at 600 nm [OD_600_] of 0.3 to 0.4 or ~10^8^ cfu/ml). Bacteria were harvested by centrifugation, and each sample was resuspended in 5 ml of phosphate buffered saline with gelatin, calcium, and magnesium (PBSG) (137 mM NaCl, 2.7 mM KCl, 4.3 mM NaHPO_4_, 1.4 mM KH_2_PO_4_, 0.125 mM CaCl_2_, 0.5 mM MgCl_2_, and 0.1% gelatin, pH 7.3). Aliquots of the suspensions were diluted and plated to confirm the starting number of bacteria. A volume of 5 ml of each culture suspension (total, ~10^9^ cfu each of wild type and mutant) was placed in the nebulizer of an Inhalation Exposure System (Model 099C A4212; Glas-Col, Terre Haute, IN). The equipment settings were as follows: 10 minutes of preheating, 40 minutes of nebulization, 30 minutes of cloud decay, 10 minutes of decontamination, vacuum flow meter at 60 ft^3^/hour, and compressed airflow meter at 10 ft^3^/hour. BALB/c mice (10 per group) were placed in the chamber during this time.

At 3 hours post challenge, the mice were euthanized by inhalation of isoflurane. Lungs were harvested and homogenized on ice in 5 ml of PBSG using a tissue homogenizer. Because were mice in a narrow age range, lungs were all similar in size. Thus, placing lungs in identical volumes for homogenization, results in standardized, reproducible results (19,28). Aliquots (50 μl) of each lung homogenate were plated on chocolate agar, and a second aliquot was plated on chocolate agar containing 15 μg/ml of ribostamycin and incubated at 35°C with 5% CO_2_ overnight; each aliquot was plated in duplicate. Colonies were counted the following day to determine the concentration of bacteria in the lungs at 3 hours after aerosol challenge. The number of colonies on the ribostamycin plates was used to calculate the concentration of the mutant. The number of colonies on the ribostamycin plate was subtracted from the number of colonies on plates with no antibiotic to calculate the concentration of wild-type bacteria in lungs. Statistical significance was determined by performing a two-tailed t test. A P value of <0.05 was considered significant. Two or three independent pulmonary challenge experiments were performed with each mutant.

## Results

### Identification of SBPs of ABC transporters

A genome mining approach to identify putative vaccine antigens led to the identification of three SBPs of ABC transporters as promising vaccine antigens for *M*. *catarrhalis*: oligopeptide permease A (OppA) substrate binding protein 2 (SBP2) and CysP. [6–8]. Guided by these observations, we conducted a more comprehensive characterization of the SBPs of ABC transporters. Table 1 shows the SBPs identified by annotation of the *M*. *catarrhalis* genome [26] along with the molecular mass of each and the ligand that is transported by the SBP. To begin to understand the potential role of these SBPs in infection caused by *M*. *catarrhalis*, we generated knockout mutants of each and assessed the mutants in parallel with the wild type strain in growth characteristics, the capacity to adhere to and invade human respiratory epithelial cells and their capacity to persist in the lungs in a mouse pulmonary clearance model.

**Table 1:** Summary of substrate binding proteins of ABC transporter systems annotated in the *M*. *catarrhalis* genome.

ABC trans-porter	SBP	SBP Mol Mass (kDa)	Lipoprotein	Putative Ligand
*opp*	OppA	72.6	Yes	oligopeptides
*sbp123*	SBP 1	28.3	Yes	lysine
	SBP 2	28.9	Yes	arginine
	SBP 3	28.6	Yes	ornithine
*met*	MetQ	29.6	Yes	methionine
*bcaa*	BCAA-SBP 1	31.3	Yes	branched chain amino acids
	BCAA-SBP 2	32.9	Yes	branched chain amino acids
*polysbp*	PolySBP	34.2	No	polyamines
*lol*	LolA	18.8	No	lipoproteins
	LolB	17.4	Yes	lipoproteins
*cys*	CysP	39.4	Yes	sulfate, thiosulfate
*znu*	ZnuA	26.1	No	zinc
*mod*	ModA	22.9	No	molybdate
*pst*	PstS	39.6	Yes	phosphate
*nrt*	NrtA	47.1	No	nitrate
*fbp*	FbpA	32.7	No	ferric ion
*ccm*	CcmE	17.4	No	heme
*afe*	AfeA	28.9	Yes	ferric ion
*unk*	UnkA	30.4	Yes	unknown

For each of the ABC transporters in Table 1, we set out to generate a knockout mutant of the gene that encodes the SBP(s) in the gene cluster and also a knockout mutant of the entire gene cluster that encodes that ABC transporter. In the case of 7 transporters, we successfully generated two independent mutants: an SBP knockout(s) and a knockout of the entire gene cluster (*opp*, *sbp123*, *znu*, *nrt*, *unk*, *mod*, *bcaa*). In the case of three transporter gene clusters (*met*, *pst*, *afe*), a knockout of the entire gene cluster was obtained. In the case of 3 ABC transporter gene clusters (*polySBP*, *lol*, *cys*), an SBP knockout was obtained but a knockout of the entire gene cluster was not because the *sbp* gene was in a region of the genome separate from the permease and ATPase genes (*cysP*) or a knockout of the ABC gene cluster was apparently lethal (*polysbp*, *lol*). In the case of two of the predicted ABC transporters (*fbp* and *ccm*), we were unable to generate either mutant (SBP or gene cluster) in spite of several attempts, presumably because these were lethal mutations. Thus, 22 knockout mutants of 13 ABC transporter systems in the *M*. *catarrhalis* genome were generated and characterized.

Note that *oppA*, *sbp123*, *cysP* and *znu* are the subject of separate publications [7, 8, 19, 28]. Selected results from these studies are included in tables with the entire set of SBPs to present a more complete view of *M*. *catarrhalis* ABC transporters; primary data of the newly characterized ABC transporters systems are reported in the present study.

### Role of ABC Transporters in Bacterial Growth

**Fig 1:**
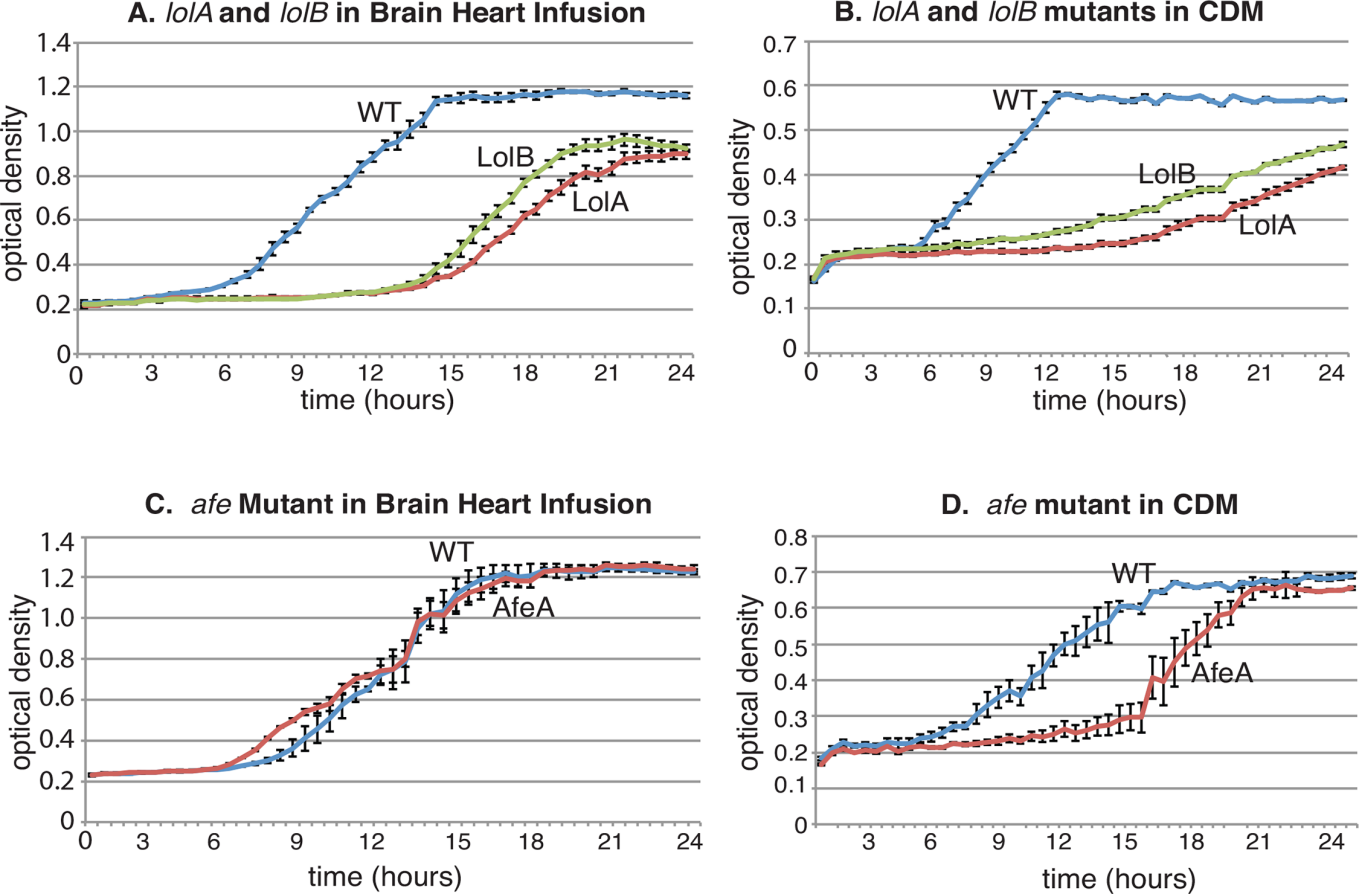
Legend. Growth curve results of *M*. *catarrhalis* wild type strain O35E and selected knockout mutants. X-axis: time (hours). Y-axis: optical density at 600 nm. The *lolA* and *lolB* mutants show slow growth (prolonged lag phase and lower growth rate) and reduced growth (lower final optical density) compared to wild type in both nutrient-rich brain heart infusion broth (panel A) and nutrient-limited chemically defined media (CDM) (panel B). The *afe* mutant has growth characteristics similar to those of the wild type strain in nutrient-rich brain heart infusion broth (panel C), whereas the *afe* mutant shows slower growth (prolonged lag phase and lower growth rate) in nutrient-limited CDM (panel D). Each point is the average of five wells, and error bars indicate standard deviations.

To assess the role of SBPs and ABC transporter systems in growth of *M*. *catarrhalis* in vitro, each knockout mutant was assessed relative to wild type for growth in BHI broth, a nutrient-rich media, and CDM, a nutrient-limited chemically defined media. Fig 1 shows characteristic growth curves of 3 mutants that demonstrate “slow” and “reduced” growth. Fig 1A and 1B shows that knockout mutants of *lolA* and *lolB*, show slower growth compared to wild type and also show reduced growth, reflected by the observation that the culture does not reach the same OD_600_ of the final culture as wild type. The Lol ABC transporter system functions in transporting lipoproteins to the outer membrane [31].

Growth of the *afe* mutant is identical to wild type in nutrient-rich media (Fig 1C). Fig 1D shows that the *afe* knockout mutant demonstrates slower growth in CDM but eventually reaches the same OD_600_ as wild type. The Afe ABC transporter functions in iron transport [32].

Table 2 shows that 15 of the 22 mutants showed identical growth in vitro in both nutrient-rich and nutrient-limited media. In addition to altered growth of *lolA*, *lolB* (lipoprotein transporters) and *afe* (iron transporter) knockout mutants (Fig 1), the zinc ABC transporter, encoded by *znu*, and the arginine transporter encoded by *sbp2* showed altered growth compared to wild type [7, 28, 33]. The *cysP* knockout mutant (sulfate and thiosulfate transporter) showed the interesting feature of slower growth compared to wild type in nutrient-rich media but identical growth compared to wild type in nutrient-limited [8]. We conclude that six SBPs of ABC transporter systems (SBP2, LolA, LolB, CysP, ZnuA, and AfeA) play a role in growth of *M*. *catarrhalis* under the in vitro conditions of these assays.

**Table 2:** Growth characteristics of knockout mutants of substrate binding proteins and ABC transporter gene clusters.

Knockout Mutant	Growth in Brain Heart Infusion Broth	Growth in Chemically Defined Media
*oppA*^a^	Full^b^	Full
*opp* gene cluster^a^	Full	Full
*sbp1*	Full	Full
*sbp2*	Slow and Reduced^c^	Full
*sbp3*	Full	Full
*met* gene cluster	Full	Full
*bcaasbp12*	Full	Full
*bcaa* gene cluster	Full	Full
*polysbp*	Full	Full
*lolA*	Slow and Reduced	Slow and Reduced
*lolB*	Slow and Reduced	Slow and Reduced
*cysP*^a^	Slow^d^	Full
*znuA*^a^	Slow	Slow
*znu* gene cluster^a^	Slow and Reduced	Slow and Reduced
*modA*	Full	Full
*mod* gene cluster	Full	Full
*pst* gene cluster	Full	Full
*nrtA*	Full	Full
*nrt* gene cluster	Full	Full
*afe* gene cluster	Full	Slow
*unkA*	Full	Full
*unkA* gene cluster	Full	Full

^a^Growth characteristics published previously

^b^Full: Full growth of mutant that is no different from wild type

^c^Slow and Reduced: Growth of mutant is slower than wild type and is reduced such that the mutant does not reach the same optical density as wild type. See Fig 1A as example.

^d^Slow: Growth of mutant is slower than wild type but eventually reaches the same optical density as wild type. See Fig 1D as example.

### Role of ABC Transporters in Adherence and Invasion of Respiratory Epithelial Cells

*M*. *catarrhalis* persists in the human respiratory tract by adhering to the epithelial surface and by invading cells. Intracellular bacteria act as a reservoir of *M*. *catarrhalis* in the human respiratory tract [34]. To assess the role of ABC transporters in adherence to and invasion of human respiratory epithelial cells, quantitative adherence and invasion assays were performed using the type II alveolar cell line A549.

**Fig 2:**
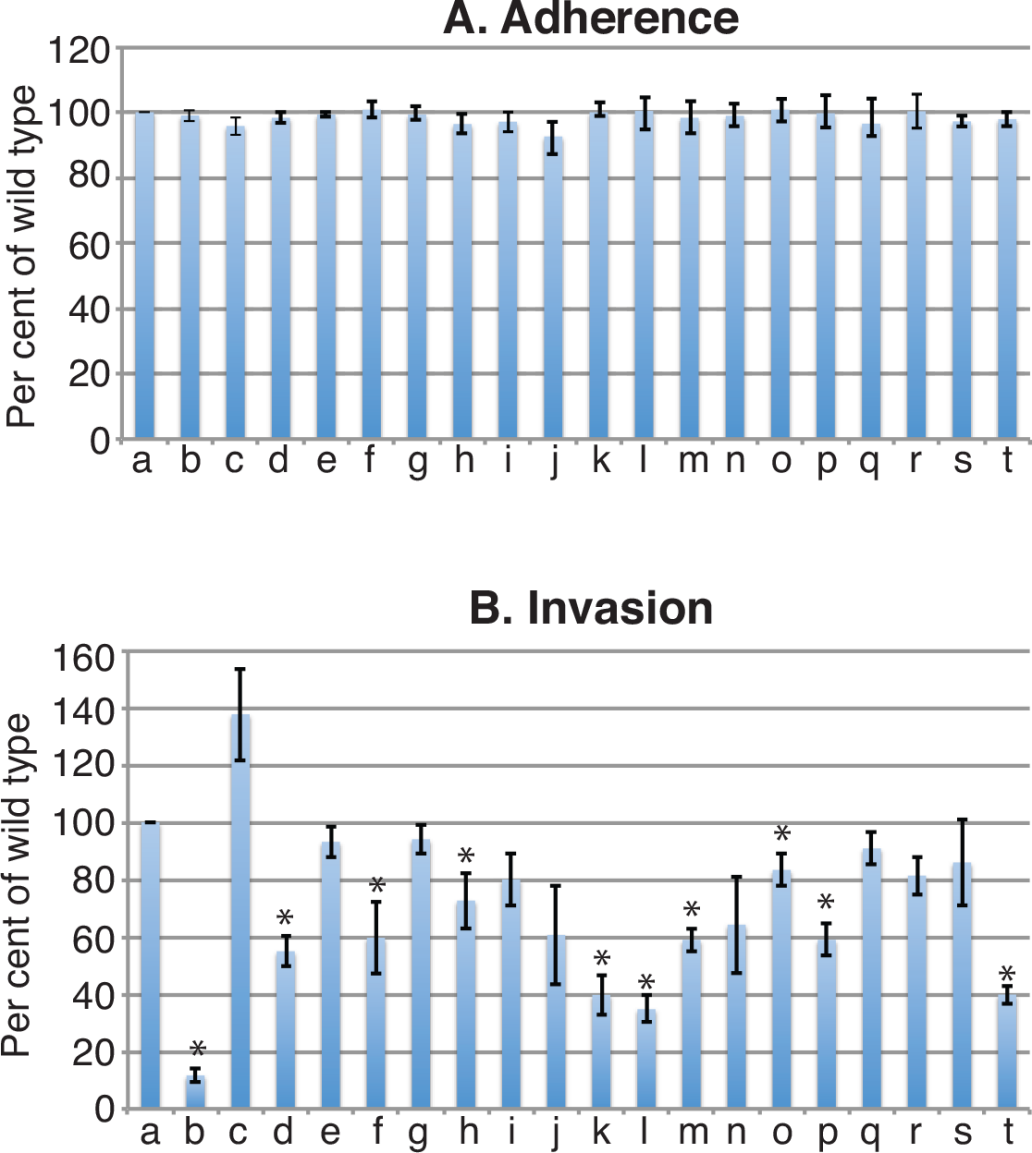
Legend. Results of adherence and invasion assays with human respiratory epithelial A549 cells. Y-axis: adherence and invasion shown as a percentage of wild type values. X-axis a) wild type; knockout mutants of: b) ***znu*** c) *znuA*; d) ***mod***; e) *modA*; f) ***unk***; g) *unkA*; h) ***cysP***; i) *pst*; j) *met*; k) ***sbp1***; l) ***sbp3***; m) ***nrt***; n) *nrtA*; o) ***oppA***; p) ***opp***; q) *bcaasbp12*; r) *bcaa*; s) *polysbp*; t) ***afe***. Error bars show standard deviations of three independent experiments. Asterisk indicates p< 0.05 compared to results for the wild type (two-tailed t test). Mutants noted in bold show significantly reduced invasion compared to wild type.

Fig 2A shows that adherence to A549 cells is identical to wild type for each mutant tested. It was not possible to test *lolA*, *lolB* and *sbp2* knockout mutants in adherence and invasion assays because the manipulation required to perform the assays resulted in loss of culture viability due to markedly impaired growth of the mutants. Based on the results shown in Fig 2A, we conclude that substrate binding proteins and ABC transporters do not play an important role in adherence to human respiratory epithelial cells in this assay system.

Fig 2B shows that 10 of the 19 mutants tested showed statistically significant reduced invasion of epithelial cells compared to wild type. Of the 10 mutants in which the entire ABC transporter gene cluster was knocked out, 5 (*znu* [zinc], *mod* (molybdate], *unk* [unknown substrate], *opp* [peptides], *afe* [iron]) showed reduce invasion. Of the 9 mutants in which genes that encode SBPs were knocked out, 4 (*cysP* [sulfate and thiosulfate], *sbp1* [lysine], *sbp3* [ornithine], *oppA* [peptides]) showed reduced invasion. These observations have important implications in how *M*. *catarrhalis* survives and persists in the human respiratory tract and are considered in the Discussion.

### Role of ABC Transporters in Persistence in the Murine Respiratory Tract

**Fig 3:**
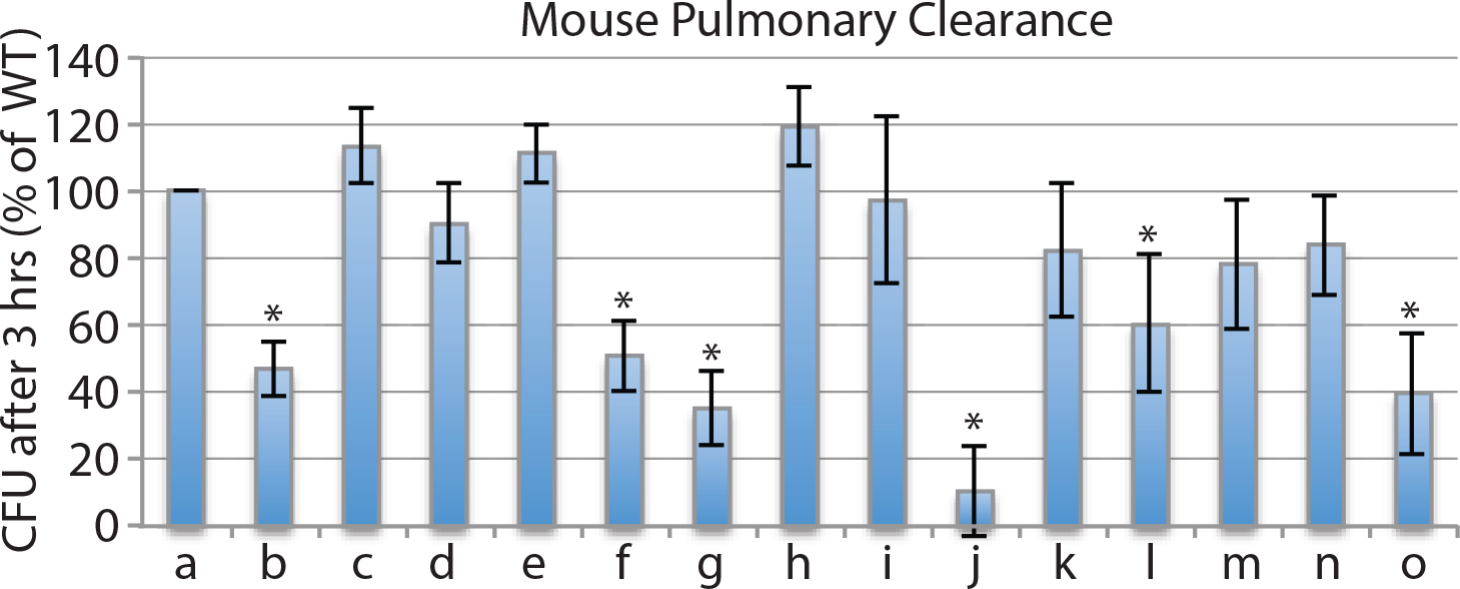
Legend. Results of pulmonary clearance in mice following simultaneous aerosol challenge by equal numbers (10^9^ cfu) of *M*. *catarrhalis* strain 035E (wild type) and individual knockout mutants. Y-axis is cfu per ml in homogenized lung tissue 3 hours following challenge. X-axis: a) wild type strain; knockout mutants of b) ***oppA***; c) *sbp1*; d) *sbp2*; e) *sbp3*; f) ***met***; g) ***bcaa***; h) *polysbp*; i) *cysP*; j) ***znu***; k) *mod*; l) ***pst***; m) *nrt*; n) *afe*; o) ***unk***. Results are the averages of 10 animals per group; error bars show standard deviations. Asterisks indicate clearance of mutant is significantly lower (p<0.05) compared to wild type (Student’s t test). Mutants noted in bold show significantly reduced persistence in murine lungs compared to wild type.

We assessed the role of ABC transporters in persistence in the respiratory tract using the mouse pulmonary clearance model. Following aerosol challenge of groups of 10 mice with equal concentrations of the *M*. *catarrhalis* wild type strain O35E and the mutant being evaluated (10^9^ cfu of each), the concentration of each strain was determined 3 hours after challenge. We tested the gene cluster knockout mutants when they were available for an ABC transporter. For those which we were unable to knock out the entire gene cluster, we tested the SBP knockout of that transporter. Fig 3 shows that 6 of 14 mutants tested showed significantly faster clearance from murine lungs compared to wild type. These included 3 transporters of amino acids or peptides (*oppA* [peptides], *met* [methionine], and *bcaa* [branched chain amino acids]; 2 transporters of ions *znu* [zinc] and *pst* [phosphate]; and one transporter of an unknown substrate (*unk*). We conclude that these ABC transporters play a role in persistence of *M*. *catarrhalis* in the murine respiratory tract.

## Discussion

In this manuscript we report the first comprehensive assessment of ABC transporters of the human respiratory tract pathogen, *M*. *catarrhalis*. Guided by the mechanisms of pathogenesis used by *M*. *catarrhalis*, the present study focused on three broad virulence properties: 1) growth and survival in nutrient-limited and hostile conditions such as those of the human respiratory tract, 2) invasion of human respiratory epithelial cells and 3) persistence in the respiratory tract in a murine pulmonary clearance model.

The first step in the pathogenesis of *M*. *catarrhalis* infection is colonization of the human nasopharynx, which is common in infancy and childhood [1, 2, 35–38]. In the case of otitis media, *M*. *catarrhalis* migrates from the nasopharynx to the middle ear where it multiplies and causes inflammation [1]. In the case of infection in adults with COPD, the bacterium infects the lower airways of the respiratory tract. Thus, as an exclusively human pathogen, *M*. *catarrhalis* has mechanisms to survive in multiple environmental niches in the human respiratory tract. It must survive at an air liquid interface in a nutritionally limited environment that varies over the course of colonization and infection. In the present study, we show that six ABC transporters play a role in growth in nutritionally limited conditions (Fig 1 and Table 2).

When *M*. *catarrhalis* colonizes the human respiratory tract, it adheres to mucin which coats the epithelial cell layer, adheres to epithelial cells, and invades cells, including epithelial cells and lymphoid cells [34, 39–41]. These intracellular bacteria act as a reservoir of *M*. *catarrhalis* in the respiratory tract, indicating that the capacity of *M*. *catarrhalis* to invade and survive inside cells of the human respiratory tract is a virulence factor. We assessed ABC transporters by studying wild type and mutant strains for their capacity to invade A549 cells. The results showed that 9 ABC transporters contribute to invasion of human respiratory epithelial cells (Fig 2).

We used the mouse pulmonary clearance model as a quantitative, in vivo model to assess wild type and ABC transporter mutant strains for their capacity to persist in the respiratory tract (Fig 3). Six ABC transporters play a role in persistence in the lungs in this model.

**Fig 4:**
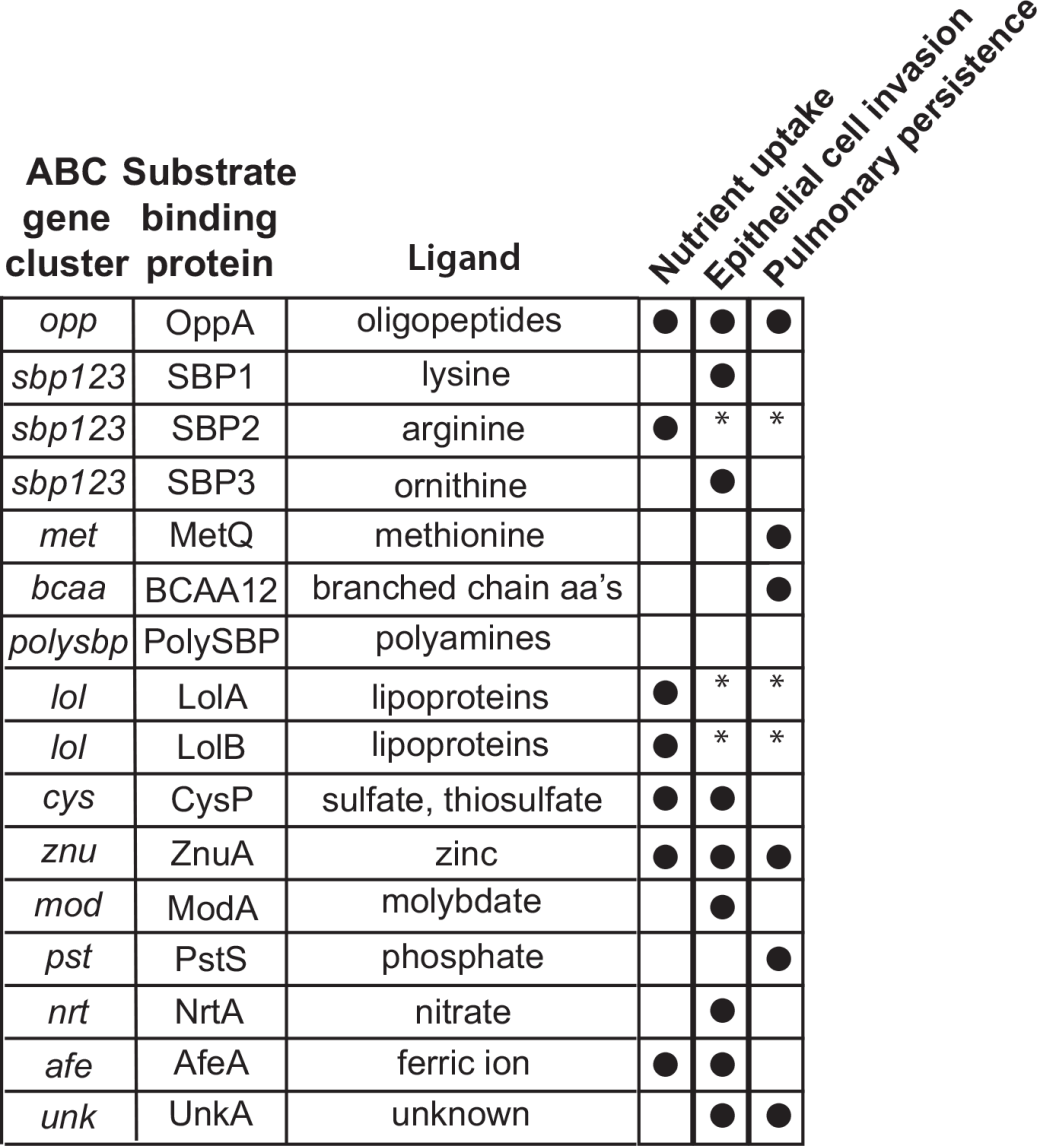
legend. Diagram summarizing the results of assays of knockout mutants in nutrient uptake, human respiratory epithelial cell invasion and persistence in the mouse pulmonary clearance model noted by columns at the right. A closed circle indicates that the result of the assay with the mutant was statistically different from that of wild type. An asterisk indicates that the assay was not possible because the mutant did not reliably grow in the conditions of the assay.

Fig 4 presents a summary of results for ABC transporter systems assessed in each of these three model systems. Overall, knockout mutants of 6 of 7 ABC transporters that function in the uptake of peptides or amino acids and all 6 knockout mutants of ABC transporters that function in the uptake of ions showed altered activity compared to wild type in at least one of the model systems tested. The various ABC transporters showed different patterns of activity, consistent with the conclusion that different SBPs and ABC transporters function at different stages in the pathogenesis of infection. For example, the lysine transporter SBP1 is important for invasion of respiratory epithelial cells but does not play a role in persistence in the mouse pulmonary clearance model. By contrast, the methionine transporter MetQ shows the opposite effect in that MetQ functions to facilitate persistence in the pulmonary clearance model but plays no role in epithelial cell invasion. Overall, the results support the concept that ABC transporters are nutritional virulence factors, functioning to support the survival of *M catarrhalis* in the diverse microenvironments of the respiratory tract.

A limitation of the present study is the drawbacks of the model systems to study the pathogenesis of *M*. *catarrhalis*. While the assays used in this study test functions that are important in pathogenesis, none of the assays completely reproduce the conditions exactly as they exist in the human respiratory tract. As an exclusively human pathogen, no good animal model systems that parallel human disease by *M*. *catarrhalis* have yet been developed. A widely used model to assess persistence of *M*. *catarrhalis* in the respiratory tract is the mouse pulmonary clearance model. The model has the limitation that *M*. *catarrhalis* does not colonize the mouse for extended periods; however, this in vivo model is reproducible, is used by multiple research groups, and has been used extensively to assess putative vaccine antigens [42–47].

Another limitation of the present study is that we may have missed some ABC transporters because we relied on annotation of the genome to identify the genes to study. In addition, it was not possible to study each mutant in all three model systems because viability of selected mutants under the conditions of the assays limited the use of some models for some mutants. For example the *lol* knockout mutants likely had structural impairments because the genes encode lipoprotein outer membrane localization pathway machinery, creating slow growth and reduced viability in several assays.

In addition, SBPs of some of the *M*. *catarrhalis* ABC transporters likely function with more than one transporter and some SBPs function exclusively with their associated transporter. We did not perform the detailed growth studies that are required to draw such conclusions.

Finally, because experiments in the present study did not include complementation of each mutation, we cannot exclude the possibility of downstream effects of the deleted genes. Guided by the results of the present study, more detailed studies of selected ABC transporters should include complementation of mutations.

In view of the importance of ABC transporters in multiple cellular functions of bacteria, these molecules have potential therapeutic value as potential drug targets. In addition to uptake of nutrients, ABC transporters participate in bacterial chemotaxis, transduction signals from ligand binding to chemosensory apparatus and binding of transcription factors [9, 48, 49]. For example, the observation that selected SBPs play important roles in facilitating persistence of *M*. *catarrhalis* in the respiratory tract make that molecule a potential valuable drug or vaccine target. Further study of ABC transporters identified in the present study represent fertile areas for drug development.
